# Quantifying SARS‐CoV‐2 Infection Risk Within the Google/Apple Exposure Notification Framework to Inform Quarantine Recommendations

**DOI:** 10.1111/risa.13768

**Published:** 2021-06-21

**Authors:** Amanda M. Wilson, Nathan Aviles, James I. Petrie, Paloma I. Beamer, Zsombor Szabo, Michelle Xie, Janet McIllece, Yijie Chen, Young‐Jun Son, Sameer Halai, Tina White, Kacey C. Ernst, Joanna Masel

**Affiliations:** ^1^ Mel & Enid Zuckerman College of Public Health University of Arizona 1295 N Martin Ave Tucson AZ 85724 USA; ^2^ Rocky Mountain Center for Occupational and Environmental Health University of Utah 391 Chipeta Way suite c Salt Lake City UT 84108 USA; ^3^ Graduate Interdisciplinary Program in Statistics University of Arizona 617 N. Santa Rita Ave. Tucson AZ 85721 USA; ^4^ WeHealth Solutions PBC 5325 Elkhorn Blvd # 7011 Sacramento CA 95842 USA; ^5^ Applied Mathematics University of Waterloo 200 University Ave. W Waterloo Ontario N2L 3G1 Canada; ^6^ Covid Watch (affiliation at time of writing, now dissolved); ^7^ World Wide Technology 1 World Wide Way St. Louis MO 63146 USA; ^8^ Systems and Industrial Engineering University of Arizona 1127 E. James E. Rogers Way Tucson AZ 85721 USA; ^9^ Ecology & Evolutionary Biology University of Arizona 1041 E Lowell St Tucson AZ 85721 USA

**Keywords:** Bluetooth technology, COVID‐19, digital contact tracing, proximity sensing

## Abstract

Most early Bluetooth‐based exposure notification apps use three binary classifications to recommend quarantine following SARS‐CoV‐2 exposure: a window of infectiousness in the transmitter, ≥15 minutes duration, and Bluetooth attenuation below a threshold. However, Bluetooth attenuation is not a reliable measure of distance, and infection risk is not a binary function of distance, nor duration, nor timing. We model uncertainty in the shape and orientation of an exhaled virus‐containing plume and in inhalation parameters, and measure uncertainty in distance as a function of Bluetooth attenuation. We calculate expected dose by combining this with estimated infectiousness based on timing relative to symptom onset. We calibrate an exponential dose–response curve based on infection probabilities of household contacts. The probability of current or future infectiousness, conditioned on how long postexposure an exposed individual has been symptom‐free, decreases during quarantine, with shape determined by incubation periods, proportion of asymptomatic cases, and asymptomatic shedding durations. It can be adjusted for negative test results using Bayes' theorem. We capture a 10‐fold range of risk using six infectiousness values, 11‐fold range using three Bluetooth attenuation bins, ∼sixfold range from exposure duration given the 30 minute duration cap imposed by the Google/Apple v1.1, and ∼11‐fold between the beginning and end of 14 day quarantine. Public health authorities can either set a threshold on initial infection risk to determine 14‐day quarantine onset, or on the conditional probability of current and future infectiousness conditions to determine both quarantine and duration.

## 1 INTRODUCTION

Manual contact tracing followed by quarantine of known contacts is a critical method for containing or mitigating the spread of communicable diseases (Armbruster & Brandeau, 2007). It is, however, extremely resource and time‐intensive and relies on case recall of contacts. New technologies can supplement this approach. Manual contact tracing can be effective for COVID‐19 (Aleta et al., 2020; Bi et al., 2020; Fetzer & Graeber, 2020; Kendall et al., 2020; Kucharski et al., 2020), however, a significant challenge is the extremely short window of time between an infected individual presenting for testing and the contacts that they infected beginning to shed infectious virus (Ferretti, Wymant et al., 2020; Kretzschmar et al., 2020). Automatic exposure notification approaches based on Bluetooth proximity have the potential to achieve many of the benefits of contact tracing, while also providing more rapid notification, greater privacy (Fraser et al., 2020; Von Arx et al., 2020), more objective recall of contacts including those whose identity is unknown to the case, and greater scalability (Ferretti, Wymant et al., 2020; Salathé et al., 2020). The two approaches of contact tracing and exposure notifications are complementary and may directly interact for example when those receiving digital exposure notifications are referred to human contact tracers for the information and support needed for quarantine adherence and further investigation (Webster et al., 2020). Exposure notification apps significantly reduced SARS‐CoV‐2 transmission in the United Kingdom, to a degree that depended on the accuracy of risk assessment (Wymant et al., 2021).

Apps have access to data on timing, duration, and Bluetooth attenuation. Determining the threshold for entering quarantine based on probability of infection should yield better results than from combining three binary thresholds for duration, distance, and the infectious period of the transmitter. A threshold for exiting quarantine based on the conditional probability of current or future infectiousness could also be used. Both would help optimize the reduction in disease transmission per day of quarantine recommended.

**Fig 1:**
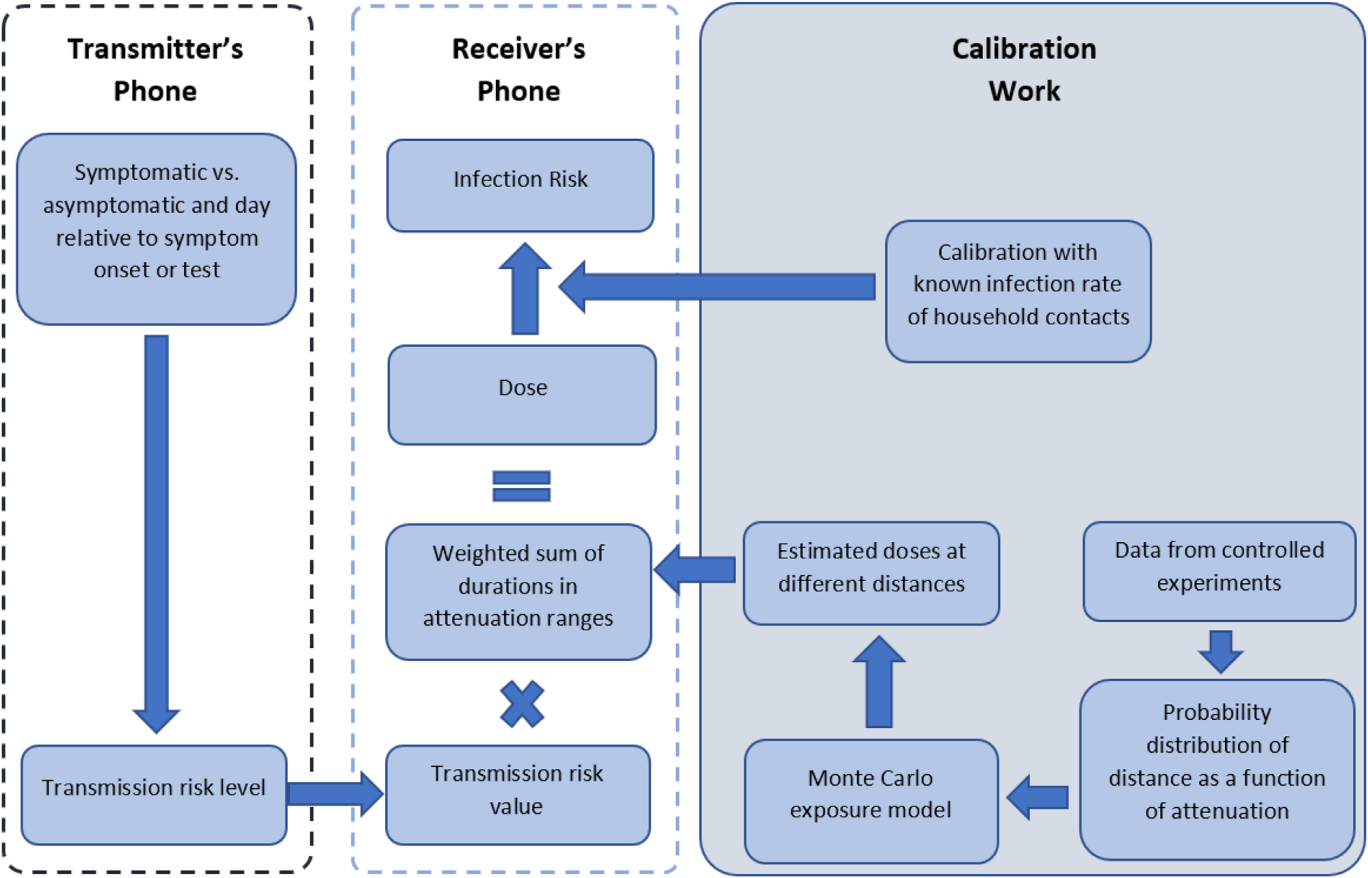
Assessment of the probability of infection following a single exposure. The calibration work is reported in this manuscript, and the procedures on the Transmitter's and Receiver's phones are part of the Covid Watch app. The terms “Transmission risk level” and “Transmission risk value” are as used in GAEN v1. In GAEN v2, it is necessary to repurpose “report type” metadata associated with Temporary Exposure Keys and combine it with the two provided levels of “infectiousness” in order to obtain up to 8 levels of infectiousness (Klingbeil, 2020a, 2020b).

Here we lay out a framework for doing so using the decentralized protocol of the Google/Apple Exposure Notification (GAEN) Application Programming Interface (API). When a user reports positive infection status, the GAEN framework (Fig. 1) allows apps to assign a “Transmission Risk Level” (version 1) or “infectiousness” (version 2) to each day that they might have been shedding, and to communicate this level to the receiver's phone via a Temporary Exposure Key (TEK). On the receiver's device, the GAEN framework records Bluetooth attenuation as a rough estimate of distance, and the duration of exposure.

The risk of infection depends on viral dose (Haas, Rose, & Gerba, 1999), which in turn depends on the shedding rate of the infected individual, and on the duration and distance of the interaction. As days go by without onset of symptoms, the probability of future infectiousness decreases, because the probability is conditioned on lack of symptoms for an increasing stretch of time. We parameterize calculations of both probabilities using both past literature and new experiments and illustrate what different risk thresholds imply for quarantine recommendations. We have since piloted the Covid Watch app using portions of this scheme on the campus of the University of Arizona 2021.

## 2 METHODS

The overall approach to calculating infection risk is summarized in Fig. 1. Parameter values and their descriptions and sources are summarized in Supporting Information Table I for calculations performed by the app and in Supporting Information Table II for parameters we used during calibration.

### 2.1 GAEN Overview

Our experiments were performed in GAEN version 1. However, the method we used is a good simulation of what became standard in the subsequently released version 2. We calculate a weighted sum of durations at different Bluetooth attenuations, using the weights to capture the differences in expected dose (number of inhaled particles over an exposure time). We then multiply by the expected infectiousness of the transmitter, as estimated from the literature in Supporting Information Methods Section 2. While this calculation is supported in version 1, access to the necessary data triggers operating system notifications of exposure even when that exposure is minimal, and so is impractical in the field. This calculation became standard in GAEN version 2, in which the dose is referred to in units of “Meaningful Exposure Minutes.” When distance changes over time, accuracy is constrained by the frequency with which GAEN records Bluetooth.

### 2.2 Experiments on Distance Attenuation Relationship

We measured Bluetooth attenuation for a range of distances, phones, and scenarios of possible signal interference with the potential to affect the attenuation—distance relationship (Supporting Information Section 1) (Farrell & Leith, 2020). Using a developer version of the Covid Watch app, we called the API multiple times with different attenuation thresholds in order to achieve resolution of 3dB in the 30dB–99dB range. The API appears to round up durations to 5‐minute increments, each with its own attenuation value; we consider each of these to be a datapoint.

Our tests were all short, for example a 12‐minute test would yield three datapoints. This is because the GAEN version 1 framework, under which the experiments were performed, records exposure durations only up to 30 minutes, in order to protect anonymity of COVID‐positive patients by limiting the risk that users will be able guess the source of their exposure, while still meeting contact definitions that invoke minimum exposure duration of 15 minutes. This cap has been lifted in GAEN version 2.

There were seven testers and 14 phones, representing a variety of models, all of iPhones—handset type and orientation can affect signal (Farrell & Leith, 2020). Forty‐nine measurements were taken with specific phone orientations, while for the remaining 986 measurements the devices were side‐by‐side and facing upwards if not otherwise specified by the barrier type (e.g., pocket). Note that 203, 222, 199, 374, 17, 20 measurements were at 0.5 m, 1 m, 1.5 m, 2 m, 3 m, and 5 m, respectively. We also used the 28, 28, 29, 27, and 16 zero‐risk barrier measurements at 0.5 m, 1 m, 1.5 m, 2m, and “N/A,” respectively. The phones were stationary during all measurements.

Note that 1,163 out of the total 1,558 datapoints were used for attenuation weight and attenuation threshold setting, with exclusions of data described in Fig. S1. The 1,163 nonexcluded datapoints are supplied in Supporting Information data set 1. Of the included 1035 attenuation measures that involved infection risk, 747 did not include a deliberate barrier, while 288 includes barriers such as pockets, backpacks, nearby laptop, and human body. Nine hundred twenty‐five measures were taken inside homes, 49 were taken inside an elevator, and 61 outside.

### 2.3 Setting Attenuation Bin Thresholds and Corresponding Weights

To rebalance the distance measurements to form a pseudo data set that is more representative of the distribution of barriers and scenarios in the real world, we created a pseudo‐data set with different multiples of the data collected at each of the distances. To inform the desired distribution of distances, we analyzed the time‐weighted pairwise distance in traffic flow simulations of a classroom (Jain, Islam, Chowdhury, Chen, & Son, 2021). These indicate a roughly uniform distribution over possible distances, with a reduction in close contact due to attempts to adhere to social distancing rules. Since close contact might be more common in other settings, and distances beyond 5m can also register Bluetooth signal, we made 5, 5, 6, 3, 132, and 168 copies of the nonzero‐risk data at distances of 0.5 m, 1 m, 1.5 m, 2 m, 3 m, and 5 m, respectively, yielding a data ratio of 1015:1100:1194:1122:2244:3360 (as a rough approximation of a target ratio of 1:1:1:1:2:3) prior to the sampling described below. To this, we added four copies of the zero‐risk barrier measurements, so that they made up 4.85% of the total pseudo‐data set. Our calibration code holds shedding rate and exposure duration constant at 50 arbitrary units/m^3^ and 30 minutes, in order to isolate the effect of distance on differences in dose between attenuation buckets.

From this pseudo data set, we first sample a datapoint that falls within the attenuation bin in question. If this is a zero‐risk barrier scenario, we assign an infection risk of 0. Otherwise, we record the distance ρ in meters. Note that our method is not based on mapping thresholds in distance to thresholds in Bluetooth attenuation, but instead on resampling from the probability distribution of distance as a function of attenuation.

We feed this distance into a microbial exposure model that estimates the airborne spread of viral particles from an emitter's mouth following a Gaussian plume formation, and their subsequent inhalation by contacts.

#### 2.3.1 Estimation of Exposure Concentrations

It is well‐acknowledged that both distance from an infected individual and duration of “close proximity interactions” (Guo et al., 2019) are important parameters in estimating the probability of infection of those exposed (Chu et al., 2020; Rea et al., 2007; Salathé et al., 2010; Setti et al., 2020). However, there is little quantitative information about the relationship between distance and risk of infection. Chu et al. (2020) quantified risk in terms of answers to binary survey questions about whether the respondent came within distance *X* of an infected person (Chu et al., 2020). They found that the value of the threshold distance X in the survey question predicts the degree to which the answer predicts risk, but this relationship cannot easily be converted into one between actual distance and risk.

For this reason, we instead model the dose inhaled at different distances. Exhaled breath is a likely source of infection (Chen, Zhang, Wei, Yen, & Li, 2020; Ma et al., 2020). Accordingly, we model a Gaussian plume (Brusca et al., 2016) of virus‐containing aerosols originating from the emitter's face at (0,0,0). The *x* axis represents the direction that the transmitter is facing and breathing toward with breath velocity *U* (m/s). Diffusion causes spread away from *y* = 0 or *z* = 0. The viral concentration is then

(1) $$C\;\left(x,y,z\right)=\frac{Q}{U}\;\frac{1}{2\pi \sigma_{y}\sigma_{z}}e^{\frac{-y^{2}}{2{\sigma_{y}}^{2}}}e^{\frac{-z^{2}}{2{\sigma_{z}}^{2}}},$$

(1.1) Q=SX,

(1.2) U=X/A,

where Q is virus emitted per second and is equal to the product of shedding rate, *S*, (in arbitrary units proportional to copies/m^3^) and an exhalation rate, *X*, (taken from measured inhalation rates in m^3^/s), yielding arbitrary units proportional to copies per second being generated (Equation 1.1). We sample our exhalation rates from a normal distribution of inhalation rates with a mean and standard deviation of 16.3 m^3^/day and 4.15 m^3^/day, respectively. These were informed by the 16–21 year old range from Table 6‐1 in the Exposure Factors Handbook (2011) (U.S. Environmental Protection Agency, 2011). To avoid negative exhalation rates, this distribution was left‐truncated at 9 m^3^/day, the smallest fifth percentile of inhalation rates for males and females in age ranges overlapping with the 16–21 year old range (U.S. Environmental Protection Agency, 2011). The velocity of breath *U* (m/s) was determined by dividing the exhalation rate (m^3^/s) by the cross‐sectional area of an open mouth A (m^2^), which is the area over which air is assumed to be exhaled at the plume source. The cross‐sectional area was informed by a uniform distribution with minimum and maximum cross‐sectional areas measured for an open mouth with a “large bite” configuration, ranging from 23 cm^2^ to 59 cm^2^ (Leckie et al., 2000). Note that for a steady‐state plume assuming a continuous output of virus, the effects of the exhalation rate (volume of air per second) on amount of virus emitted, and on the velocity with which they disperse, cancel out. For an abrupt exhalation such as a cough, rather than steady state, a higher exhalation rate would affect viral airborne concentration.

For interactions ≤1 m, we assumed two people interacting are directly in front of each other along the *x*‐axis (φ = π/2, θ = 0). For interactions beyond the close range (>1 m), we sample θ from a uniform 360 degrees (min = 0, max = 2π), and the angle between the *z* axis and the *xy*‐plane, φ, was randomly sampled from a triangular distribution (min = π/4, mode = π/2, max = 3π/4). We then convert from spherical units to (*x*,*y*,*z*) to apply Equation 1. We assumed that scenarios where the person exposed was behind the emitter (*x*<0) resulted in a zero dose.

To capture the shape of the plume, we use:

(2) $$\sigma_{y}=I_{y}\;x,$$

(3) $$\sigma_{z}=I_{z}\;x,$$

Assuming moderately stable conditions, $I_{y}$ and $I_{z}$ were randomly sampled from uniform distributions with minimums and maximums of 0.08–0.25 and 0.03–0.07, respectively (Western Engineering, n.d.).

We note that inhalation and exhalation rates are both likely important to risk. For example, one infected dance instructor spread COVID‐19 to seven of 26 other instructors at a workshop (Jang, Han, & Rhee, 2020), representing a similar risk as for household contacts, despite the presumption that most were at >2 m distance for most of this time. Limited air circulation or increased respiratory rates are important factors that cannot be captured in the current GAEN approach, but the four‐hour duration of the workshop is captured in GAEN version 2, and when combined with considerable uncertainty in the relationship between Bluetooth attenuation and infection risk per minute, this can appropriately capture the high risk of such a scenario.

While wind velocity and relative humidity are important factors for determining droplet and fine aerosol dispersion and deposition (Feng, Marchal, Sperry, & Yi, 2020; Yang, Elankumaran, & Marr, 2011), as is mask usage, these are uncertain factors that are not recorded by the app, especially considering that interactions may occur indoors or outdoors. By not accounting for deposition, and by assuming that masks are either not worn or not worn effectively, we will tend to overestimate dose at greater distances, and in the presence of masks. This will implicitly lower the app‐imposed risk tolerance of individuals who comply with public health guidelines that recommend masks and physical distancing, and who might therefore also be more inclined to comply with quarantine recommendations. The 2‐m rule was based on the assumption that most transmission is via droplets (large aerosols) for which deposition occurs over this distance. However, there is increasing evidence for transmission via smaller aerosols (Fennelly, 2020; Jones et al., 2020; Lednicky et al., 2020; Prather, Wang, & Schooley, 2020; Qureshi et al., 2020), supporting our assignment of some risk to greater distances, reflecting short‐ to medium‐distance airborne transmission.

#### 2.3.2 Inhaled Dose per Interaction

An inhaled dose of viral particles due to person‐to‐person interactions was estimated based on the duration of the interaction (minutes) (*T*), the concentration of virus in the air at this {*x*,*y*,*z*} coordinate during the interaction (arbitrary units of viral particles/m^3^) *C*(x,y,z), and inhalation rates (m^3^/minute) (I),

(4) $$D\;=\;T\cdot I\cdot C\left(x,y,z\right)$$

**Fig 2:**
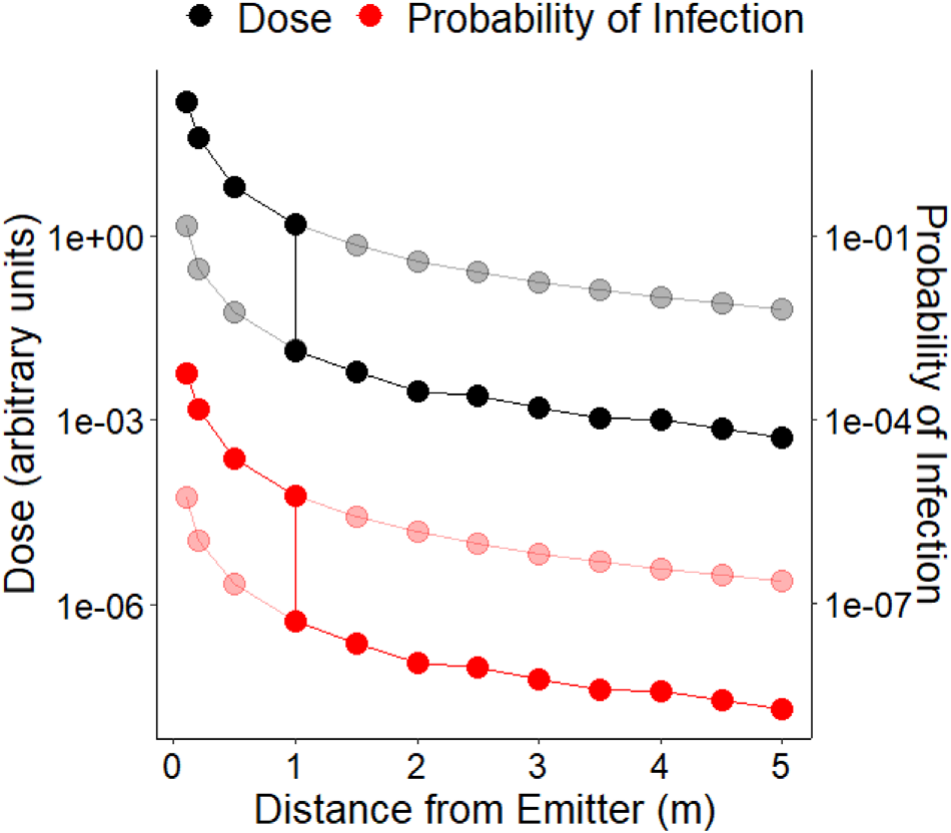
Expected dose and corresponding probability of infection for a 30‐minute exposure, estimated using our Monte Carlo procedure as a function of distance from an infected individual *The discontinuity at 1 m indicates our assumption that this distance threshold indicates face‐to‐face interaction. Faded points show doses and infection risks that would be estimated if a face‐to‐face or nonface‐to‐face interaction assumption were consistent across distances. The bolded points indicate what we assumed in our framework. Note that Bluetooth information likely contains more risk information regarding whether an interaction was face‐to‐face than it does about risk as a function of the distance at which either a face‐to‐face or a nonface‐to‐face interaction takes place. The WHO close contact definition invoking 1 m also invokes face‐to‐face interaction (World Health Organization, 2020). The same is true, only with 2 m, for European guidance (European Centre for Disease Prevention and Control, 2020) The Centers for Disease Control and Prevention (CDC)’s definition departs from this in omitting reference to face‐to‐face when referring to interactions occurring within 6 feet (Centers for Disease Control and Prevention, 2020).

Inhalation rates were randomly sampled from the same distribution as exhalation rates but allowing for a different value per iteration. As with exhalation rates, we left‐truncated the distribution to avoid negative inhalation rates and therefore negative doses. Fig. 2 shows the expected dose as a function of distance, with a discontinuity at 1 m arising from our assumption that this distance or below indicates face‐to‐face interaction.

We use a Monte Carlo approach to sample angle, exhalation rate of the transmitter, cross‐section of the transmitter's open mouth, and inhalation rate of the exposed individual, to obtain a mean dose/time for that attenuation bin. For distances ≤1 m, we assume face to face interactions, consistent with distances measured for “interpersonal” interactions (Zhang et al., 2020). We choose thresholds between attenuation bins, and relative risks for time spent in each bin.

To select the threshold values (*a*, *b*) demarcating three attenuation bins, we optimized the differences in mean dose between two randomly sampled attenuation measurements. Specifically, we maximized the value of

$$d\;\left(a,b\right)=\sqrt{2p_{A}p_{B}{\left(A-B\right)}^{2}+2p_{B}p_{C}{\left(B-C\right)}^{2}+2p_{A}p_{C}{\left(C-A\right)}^{2}}$$

where A,B, and C are the average doses D from Equation 4, averaged across Monte Carlo sampling described above, corresponding to bins [0, *a*], [*a*, *b*], and (b, +), and $p_{A}$, $p_{B}$, and $p_{C}$ are the probabilities that an attenuation will fall within that bin in our pseudo data set.

We examined multiple local maxima of this distance measure before choosing a partition pair. We also investigated alternative versions of a distance metric and alternative rebalancing schemes, to confirm that this is a relatively robust partition pair.

To relate estimated dose to infection risk, we use an exponential dose–response curve, which is derived from the assumption that each host is susceptible and that each virus has an independent probability of survival and subsequent initialization of infection (Haas et al., 1999). In our case, this probability k, multiplied by a constant C to convert from arbitrary units to number of virions, sets the parameter λ=kC in the equation

$$P\left(\mathrm{infection}\right)=\;1-e^{-\lambda D},$$

where expected dose D comes from a shedding rate multiplied by a weighted sum of time spent within three attenuation ranges. An exponential dose‐response curve is superior to the approximate beta‐Poisson for some other viruses (http://qmrawiki.org/content/recommended‐best‐fit‐parameters, accessed 09/07/2020). These viruses include adenovirus, enterovirus, poliovirus, and SARS‐CoV‐1.

### 2.4 Calibrating the Dose–Response Curve

Our weighted sum of durations and our estimates of shedding rates S in the Results are both in arbitrary units. We therefore fit λ to obtain infection probabilities that are compatible with household spread. Asymptomatic infection and low test sensitivity can both deflate estimated household infection risks, while indirect chains of infection via a third household member can inflate them. A meta‐analysis by Curmei *et al*. attempted to correct for these complications and estimated a secondary attack rate of household contacts of 30% (Curmei, Ilyas, Evans, & Steinhardt, 2020). We assumed exposure is equivalent to eight hours with the maximum shedding rate in the lowest attenuation and calculated λ for this dose that would result in a 30% infection risk. Note that during the long review process for this manuscript, significant evolution of SARS‐CoV‐2 took place, and secondary attack rates have correspondingly risen since then.

### 2.5 Probability of Current or Future Infectiousness

Our scheme can be used either to (1) set a threshold on the initial probability of infection to trigger 14‐day quarantine, or (2) set a threshold for the probability of current or future infectiousness to determine both who should quarantine and for how long. To calculate residual risk of infection as a function of initial risk plus time since exposure, we use the probability distribution of incubation periods from Lauer et al. (2020), available at https://iddynamics.jhsph.edu/apps/shiny/activemonitr/. Note that it is possible that incubation periods are even more dispersed than reported here (Wei et al., 2020); this would lengthen quarantine recommendations.

To calculate risk of current or future infectiousness, we assume a fraction of symptomatic versus asymptomatic cases and take an average of the discount factors applying in each case. Across a population, 20% of infections are estimated to be asymptomatic (Buitrago‐Garcia et al., 2020). Younger users are more likely to be asymptomatic (Davies, Klepac, Liu, Prem, & Jit, 2020), so the fraction of asymptomatic cases could be personalized on the basis of user age if that information is collected on a voluntary basis. For the symptomatic cases, we discount according to the probability of subsequently developing symptoms, given that symptoms have not appeared yet.

For the asymptomatic cases, we combine the incubation periods from (Lauer et al., 2020) with a distribution of shedding durations from (Long et al., 2020). Long et al. (2020), report slightly longer shedding durations for asymptomatic than symptomatic shedding but other studies for which we were unable to obtain the data, report the opposite, or no difference (Chau et al., 2020; Chen et al., 2020; Hu et al., 2020; Xiao et al., 2021; Yang, Gui, & Xiong, 2020). Shedding declines in magnitude post symptom onset and is considered by the Centers for Disease Control and Prevention (CDC) to have reached negligible levels by 10 days post symptom onset. We assume that asymptomatic shedding begins three days before what would have been the day of symptom onset if symptomatic, or else immediately upon infection, whichever occurs later.

Using this assumption, we calculated the probability distribution of the day that shedding ends, given both the distribution of incubation periods and a distribution of shedding durations. For the latter, we combine the asymptomatic and symptomatic shedding durations of (Long et al., 2020) but on the basis of CDC advice for isolation, we truncate the distribution so that all shedding periods longer than 12 days are recorded as exactly 12 days.

Note that low dose exposures, for example to asymptomatic individuals, may result in longer incubation periods (Wei et al., 2020), suggesting that low initial risk scores should have longer rather than the shorter quarantines we calculate using this method. We currently ignore this by assuming that risk scores primarily capture uncertainty in the likelihood of infection with a minimal dose, and not variation in the infecting dose once above the minimal. This is supported by genetic evidence in support of an extreme population bottleneck of only 1–8 virions upon transmission, despite using clinical samples data that included presumed high‐dose transmission (Lythgoe et al., 2021). We note that lognormal distributions of incubation periods with substantial variance occur even in the absence of variation in dose, due both to variance in within‐host replication rate and to the stochastics of establishing infection in the first cells (Ottino‐Loffler, Scott, & Strogatz, 2017).

To see how the assumption of negligible variance in infecting dose arises from our model, note that the exponential dose–response curve we use assumes that each virion has an independent probability of initiating infection. Under the resulting Poisson distribution for the number of virions responsible for the initial infection, then even for the 30% infection rate of household contacts, the probability that infection is initiated with two or more virions is only 5%, and with three or more virions is only 0.6%.

However, the higher variance in dose explored in Supporting Information Fig. S2 could make initiation with multiple virions common enough to matter for high infection probabilities. In this case, our simplifying assumption might require overly long quarantines following very high risk exposures. Unless the variance is extreme, it will not significantly distort estimated probabilities among the range of lower risk exposures.

### 2.6 Negative Test Results to Shorten Quarantine

This method can be extended to include the effect of a negative test result on a recommended duration of quarantine. Incorporation of negative test results can help exclude asymptomatic infection and hence allow for earlier release. From Bayes Theorem, and taking the false positive rate as negligible, a negative test result changes the probability of infection from p to $\frac{Ep}{\left(1-(1-E)p\right)}$, where E is the false negative rate. This could be taken as 0.3 (Ai et al., 2020; Y. Yang et al., 2020) or made dependent on the timing of the test relative to exposure (Hellewell, Russell, Beale, & Kucharski, 2020; Kucirka, Lauer, Laeyendecker, Boon, & Lessler, 2020).

Kucirka et al. (2020) report a false negative rate as a function of the timing of a PCR test relative to symptom onset, but most of the data are postsymptom onset, with only a single patient's data informing false positive rates prior to symptom onset. The data of (Hellewell et al., 2020) are more suitable for combining with the distribution of incubation periods to calculate the false negative rate as a function of time since exposure, conditional on lack of symptoms to date (Petrie, Nurtay, Ferretti, Fraser, & Masel, 2021). Careful treatment of shared conditionality on symptom onset day enables such calculations even when there are exposures on multiple days (Petrie & Masel, 2021).

### 2.7 Multiple Exposures and Total Risk

Note that strictly speaking when using this latter threshold, our “quarantine” recommendations are, through their treatment of the possibility of undiagnosed asymptomatic infection, a combination of quarantine and isolation. Our scheme, by expressing exposures in terms of probabilities of infection and infectiousness, naturally lends itself to combining risks over multiple exposures. GAEN version two sums exposures over 24 periods beginning and ending at midnight UTC). To calculate total risk, we combine the probabilities $p_{i}$ of each exposure i, each discounted as described in the section above, as $1-\prod_{i}\left(1-p_{i}\right)$.

**Fig 3:**
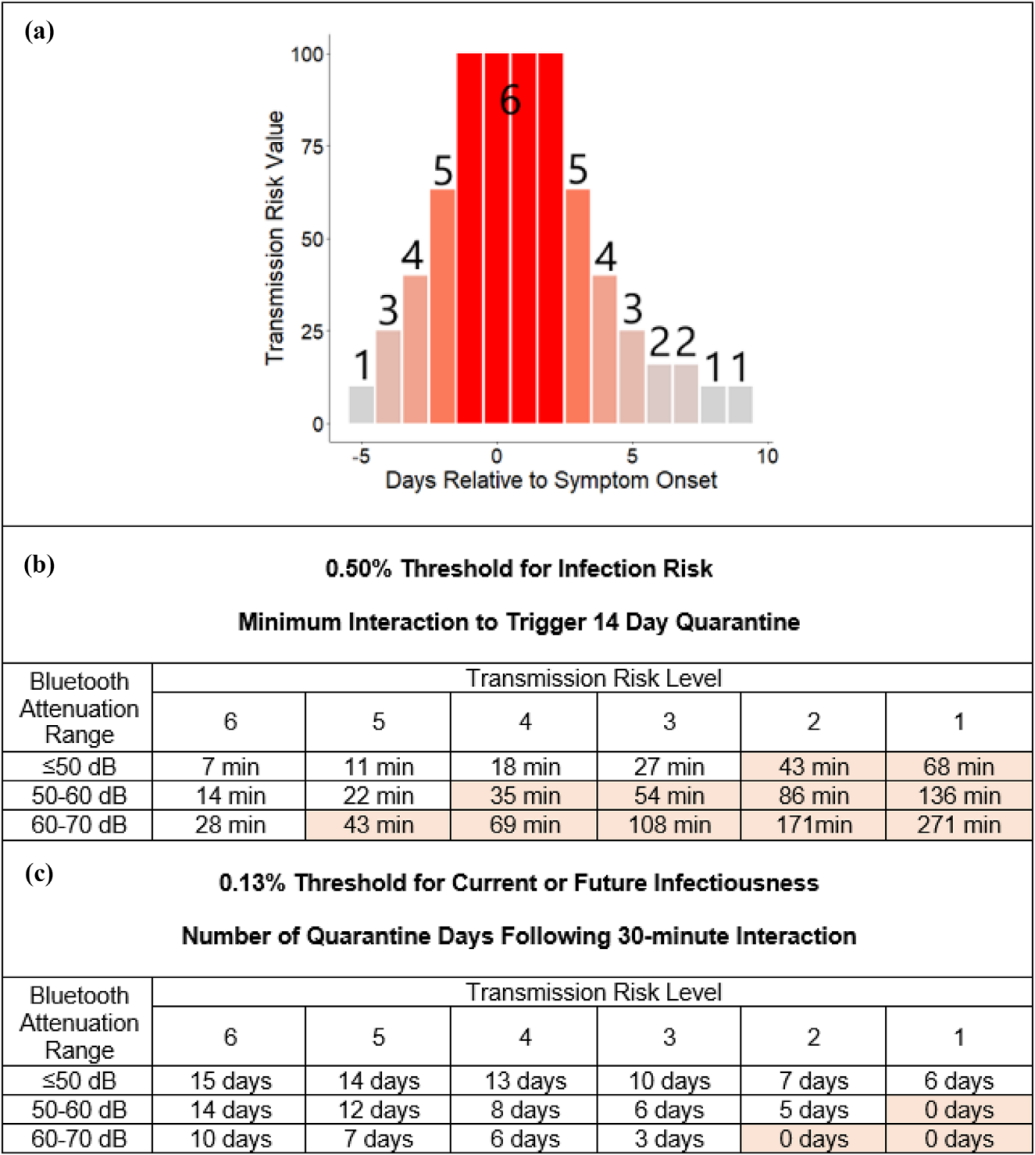
Examples of quarantine recommendations using a threshold for infection risk (B) vs. for current or future infectiousness (C). (A) Transmission risk levels 1–6 are used to capture the 10‐fold range of relative infectiousness on different days as a function of timing relative to symptom onset. Evidence from both transmission pairs and TCID50 measurements is reviewed in the Supplementary Materials Section 2. (B) The minimum length interaction needed to trigger 14‐day quarantine is a function both of Bluetooth signal attenuation and of infectiousness. Approaches that neglect the latter correspond to a single row of 15 minutes, and potentially a second row of 30 minutes. Shaded cells indicate that a 30‐minute interaction would be insufficient to trigger quarantine, creating issues for GAEN version 1. (C) Number of quarantine days recommended following a 30‐minute interaction.

**Fig 4:**
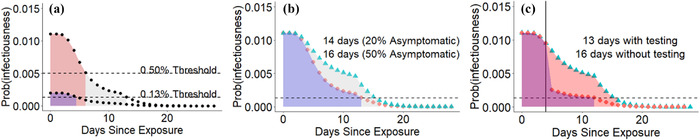
Applying a consistent risk tolerance for current or future infectiousness causes quarantine duration to be a function of initial risk, of the tolerated degree of risk, of the fraction of infections that are assumed to be asymptomatic, and of any negative test results. (A) Initial infection risk is 1.10% following 15 minutes of close contact with an individual around the time of symptom onset. With a 20% asymptomatic fraction, a 14‐day quarantine is recommended under a 0.13% risk threshold, but only a seven‐day quarantine under a 0.5% threshold. Following a lower risk exposure with 0.2% infection risk, quarantine would be 5 days with the stricter threshold, and there would be no quarantine with the less strict. (B) Quarantine must be longer to mitigate a high likelihood of asymptomatic infection in the exposed individual. (C) A negative test result, shown here as taking place on Day 5, can shorten quarantine, in particular mitigating the risk of asymptomatic infection. We apply Bayes theorem with 70% sensitivity and 100% specificity. Note that widespread availability of testing would allow much stricter risk thresholds to be used. Day 0 is included in the total quarantine times.

Figs. 3 and 4 illustrate scenarios of a single exposure. When there are multiple exposures, quarantine durations are determined with respect to total risk. The risk threshold for initiation and completion of quarantine are the same. In other words, risk is treated in an internally consistent fashion to maximize the benefit from a given number of recommended quarantine days across a population. When fixed quarantine durations are used, exposure must be significant on a single day, from which the 14 days are then calculated, and risks are not integrated across multiple days.

## 3 RESULTS

Our Gaussian plume model of microbial exposure produces the relationship between distance and infection risk shown in Fig. 2. Training on both this and our distance attenuation measurements (as summarized in Methods section 2.2), we chose attenuation bins of ≤50 dB, 50–60 dB, and 60–70 dB, with weights 2, 1, and 0.5, respectively. GAEN version 2 refers to these attenuation bins as Immediate, Near, and Medium. We assign a weight of 0 for >70 dB not because there is no residual infection risk, but because the maximum distance for which BlueTooth signals are still recorded can be highly device‐dependent.

Using these weights, we calibrate λ = 3.70 × 10^−6^ (see Methods section 2.4) to obtain an infection probability of 0.30 for household contacts. Note that the best way to calibrate both weights and λ would be after the app is rolled out, with manual contact tracers or other opt‐in data export compiling exposure characteristics and relating them to the rate of subsequent infection. While Equation 5 calculates the function of an expectation rather than an expectation of a function, treating variance in dose amounts to using an “effective” value of λ (Supporting Information Section 1).

Bluetooth attenuation thus only distinguishes a twofold difference in dose and hence risk between Immediate and Near range, and only fourfold between Immediate and Medium range. In contrast, informed both by TCID50 data (Bullard et al., 2020) and by epidemiological evidence (Ferretti, Ledda et al., 2020), we assign a 10‐fold higher risk to exposures to individuals during peak shedding than during the margins of the infectious period (Supporting Information Section 2, illustrated in Fig. 3A). The magnitude of shedding (infectiousness) has received less attention than attenuation and exposure duration. It was not widely used by other GAEN apps until version 2 pushed the use of two rather than one levels of infectiousness, determined relative to symptom onset day, and a working group was convened to recommend settings, including input from the current work (Wanger, 2020).

The relatively low predictive power of Bluetooth attenuation gives rise to diagonal patterns in the quarantine recommendations in Fig. 3B. These diagonal patterns mean that quarantine will sometimes be recommended following prolonged exposure to a high shedder, even if the interaction took place at well beyond the estimated 2 m distance. However, these exposures are not risk‐free either, in particular if taking place in an indoor environment, especially in cases with heavy breathing, such as exercise environments (Jang et al., 2020) or choir rehearsals (Hamner et al., 2020), where aerosols may mix throughout the room and also deposit on surfaces. The diagonal pattern reflects the compelling evidence that exposure timing and duration also significantly contribute to infection risk. We therefore sometimes recommend quarantine recommendation even when Bluetooth attenuation, which is a poor proxy for distance, is not low. However, Bluetooth attenuation is nevertheless critical to concluding that an interaction occurred at all.

So far, we have estimated the probability of infection from an exposure. Each day that passes without symptoms provides more information to make infection less likely, and eventually also to increase the probability that shedding from an asymptomatic infection has ended. To calculate the probability of current or future infectiousness on a subsequent day, conditional on no symptoms until that day, we apply a discount factor based both on time elapsed without symptoms and also any negative test results. We multiply the probability of infection from an exposure by this discount factor to determine the remaining risk of infectiousness from a given exposure.

Traditional quarantine guidelines are binary (either 14 days from date of last exposure, or no quarantine required). However, a consistent approach to risk, combined with a desire to impose quarantine days in the most efficient manner possible to combat disease spread, suggests that individuals should quarantine for longer following a higher‐risk exposure (Fig. 4A) (although see Section 2.5 for a caveat with very high doses). This approach calculates the number of days postinteraction that would be needed to drop below a given threshold of probability of current or future infectiousness. Exposure scenarios of 30 minutes are illustrated in Fig. 3C.

We used a 0.13% threshold in Fig. 3C, because it recommends a 14‐day quarantine for 15 minutes in close range with a high shedder. Such an interaction has a 1.10% infection risk, which falls below a 0.13% probability of current or future infectiousness after 14 days of quarantine during which no symptoms appear. Note that this initial infection risk is broadly compatible with the attack rate reported in Taiwan (1.0%, 95% CI: 0.6%–1.6%) for those interacting with infected individuals in the first 5 days of symptom onset (Cheng et al., 2020), which is similar to the 1.9% attack rate (95% CI 1.8%−2.0%) reported in South Korea (Park et al., 2020).

Current advice treats the larger risk of longer exposure the same, making a 0.13% threshold more conservative because it is calculated to generate a 14‐day quarantine for a minimal duration of exposure. However, this is offset by our assuming maximal shedding in calculating this benchmark example. In other words, while this threshold approximates the risk tolerance of current advice, the details of who is recommended for quarantine and for how long will be different in our quantification of total risk than it would be if we were to combine independent binary thresholds for infectious period of transmitters, duration of exposure, and distance to produce a quarantine duration of uniform length. This leads to more consistent treatment of risk to yield a larger benefit in terms of transmission prevented per day of quarantine recommended. Shorter quarantines might significantly reduce the harms imposed by quarantine (Brooks et al., 2020), and increase compliance (Soud et al., 2009, although see McVernon et al., 2011). Quarantining for 14 days postexposure may be exceptionally challenging for essential workers, individuals without sick leave, or those who would endure significant financial hardship due to lost income.

The assumed fraction of asymptomatic infections affects the discounting of risk. The symptomatic fraction is discounted according to the distribution of incubation periods from exposure to symptom onset, while releasing the asymptomatic fraction from quarantine is not safe until not only onset, but also significant shedding is over (Section 2.5). Our calculations so far assume that 20% of infections are asymptomatic. If we instead assume that 50% infections are asymptomatic, for example in a young age group, even a 15‐minute contact registered as low attenuation and with peak shedding in the transmitter would require a 16‐day quarantine to meet a 0.13% threshold (Fig. 4B). However, if an individual were to test negative during their quarantine, their conditional probability of current or future infectiousness would drop, shortening their quarantine to 13 days for a test with 70% sensitivity (Fig. 4C).

## 4 DISCUSSION

Here we quantify relative risk of infection using experiments to inform the noisy distance attenuation relationship, and Monte Carlo simulations to inform both this and other sources of variability and uncertainty that affect risk. We roughly calibrate relative infection risk to absolute probability of infection based on limited information from the infection probability of household contacts.

Errors in calibration are likely, but will generally not affect the rank order of risks. For example, adjusting the risk threshold of 0.13% for quarantine will have similar effects to adjusting the value of λ. Knowledge of absolute versus relative risk does have some effect once some saturation in risk begins to occur, little of which will occur unless much longer durations are recorded.

With 20% cases being asymptomatic and no testing, the risk of current or future infectiousness falls ∼11‐fold over the first 14 days of quarantine. Under GAEN v1.5, risk will sometimes differ more between two individuals entering quarantine than when comparing the same individual before vs. after a 14‐day quarantine. For this reason, our scheme could recommend quarantines longer than 14 days. Variation in quarantine length is to be expected—if total risk is scored consistently, some quarantines will be longer and others shorter, in order for residual infection probability, conditional on time elapsed without symptoms, to fall below a threshold.

The Covid Watch app is currently programmed either to use a threshold on infection risk to determine 14‐day quarantine onset, or on risk of current and future infectiousness to determine both quarantine and duration. Either threshold can be set by public health authorities flexibly in the light of external factors such as level of community transmission, jurisdictional comfort with uncertainty related to digital exposure notifications, and current public health science and recommendations. Communities that have achieved containment might choose to set a stricter threshold, testing individuals once or twice to lower their risk following each negative test. Communities with high prevalence might raise the threshold if it seems likely that the number of quarantine recommendations being issued by the app will cause it to fall out of use, although this issue has not as yet been reported.

When a threshold is set well below the probability that a randomly chosen member of the population is currently infected, it should be recognized that individuals agreeing to download and comply with the recommendations of the app are implicitly agreeing to adhere to higher standards than those implied by the current absence of a general stay‐at‐home order (Petrie & Masel, 2020). At the time of writing (January 16, 2021), the rate of current infection is ∼5% in Arizona (Gu, 2020). Note that the maximum possible initial infection risk under GAEN version 1 comes to 3.81%. GAEN version 2, by relaxing the 30‐minute cap on durations, has made possible resolution among higher risks, although it has complicated approaches to resolve levels of infectiousness.

When the infection risk of the average person in the population is high, we believe that the best solutions are population‐level restrictions and closures (Petrie & Masel, 2020). Even when such restrictions are in place, a GAEN app might still have some utility, especially for essential workers. A GAEN app could also be an inferior but still useful option should the political will for population‐level restrictions not exist.

As the conditional probability of current or future infectiousness (conditioned on the exposed individual being asymptomatic) falls throughout their quarantine period, messaging can also change. For example, during the initial high‐risk days, users might be offered concrete resources such as grocery delivery, or the option to quarantine in a specialized facility in order to protect other household members, before transitioning to self‐quarantine once risks falls. Even with self‐quarantine, an app might identify the days on which staying home is the highest priority (i.e., days where the potential infectivity may be highest). Messaging considerations are discussed in Supporting Information Section 3.

We caution that our derived relationship between Bluetooth attenuation and infection risk is extremely approximate and model‐dependent. For example, our model calibration assumes that individuals are stationary and that the distance between phones represents the distance between individuals. Additionally, we do not address risk reduction benefits of masks, since mask usage is not captured by the app. Mask filtration efficacies vary by material type, adequacy of fit, and particle size (Pan, Harb, Leng, & Marr, 2020).

Because conservatism versus permissiveness ultimately depends on the risk threshold, exclusion of mask wearing constitutes overestimation of the relative risk of masked contact, with underestimation of the relative risk of unmasked contact as a corollary. The way to make risk assessment more conservative is to decrease in the risk threshold, while keeping upstream assumptions as accurate as possible. The inability to accurately assess risk with respect to mask use is mitigated by the fact that mask‐wearing individuals are likely to have lower risk tolerance, making it more acceptable that a lower implicit risk threshold is applied to them.

Regarding uncertainties in model parameters captured by the app, we have more confidence in our settings of infectiousness levels for symptomatic cases, but very little for asymptomatic cases. These parameters need to be calibrated with real world data on app users who report their app‐recorded exposures to manual contact tracing efforts, who then track which users go on to test positive, and who are therefore able to mine the data to quantify the quantitative relationship between exposure details (duration, attenuation, infectiousness) and probability of infection. Transfer of this data to central databases, ideally contact management databases, is critical to improve the targeting of quarantine recommendations to those at highest risk of being infected. Improved risk calibration will make most efficient use of each day of quarantine recommended to reduce transmission.

Short of this, more quantitative data on infectivity would be extremely valuable. Our determination of infectiousness partly relies on the prospective sampling of all individuals in a skilled nursing facility (Arons et al., 2020), where many patients subsequently got sick. Daily samples during similar outbreaks could be used to quantify how shedding varies both among individuals and as a function of time relative to symptom onset. TCID50 data would be ideal, but even *Ct* values can be valuable for this purpose. However, the fact that the settings we originally chose based infectivity data agreed with later and improved epidemiological approaches is encouraging (Ashcroft et al., 2020; Ferretti, Ledda et al., 2020).

Without the extended durations provided in version 2 of GAEN, our default calibrations will not recommend quarantine (Fig. 3B) or extended quarantine (Fig. 3C) for contact with less infectious individuals. However, with the duration cap lifted in GAEN version 2, 43 minutes in the ≤50 dB range, 1.43 hours in the 50–60 dB range, or 2.85 hours in the 60–70 dB range with an individual of transmission risk level 2 would be sufficient to trigger quarantine (Fig. 3). However, GAEN version 2 provides only two levels of infectiousness, and implementing more as Germany has done (Klingbeil, 2020a, 2020b), requires managing the considerable complexities of separate calculations for shared key servers that need to be interoperable (Justus Benzler, personal communication).

Limited durations and infectiousness information have been driven by privacy concerns, but this must be weighed against the significant ethical considerations in favor of efficient allocation of quarantine (Singer & Masel, 2020). In Supporting Information Section 3, we suggest an alternative method to preserve anonymity, which is to conceal all exposure details from the user's view. When using variable quarantine duration, this also effectively conceals the date of exposure.

Our framework can be used not only to guide recommendations for who should quarantine and for how long, but also to allocate associated resources including quarantine facilities, grocery delivery and other social support, and priority for access to scarce tests. Both manual contact tracing and digital exposure notification require rapid testing to be effective. Given limited tests, targeting those at highest risk of infection will do the most good in finding new positive cases who are early enough in the course of infection for these approaches to stem transmission the most.

## CONFLICT OF INTEREST

JIP, ZS, SH, TW, and JM have ownership stakes in WeHealth PBC, which distributes the Covid Watch Arizona app.

## Supporting information



Supplementary Table I. Parameter values used by app to calculate risk
Supplementary Table II. Parameter values used by us to calibrate parameter values in Table 1
Fig. S1. Attenuation data cleaning
Fig. S2. Although our dose‐response curve takes the function of an expectation, for low infection probabilities the effect of this is to change the interpretation of the value of λ, which is 3.70 x 10^‐6^ when using Eq. 5 but 0.3 times this value when using Eq.6 with a distribution of log‐dose with standard deviation corresponding to a 16‐fold difference in dose.

## Data Availability

Supplementary Data Table I provides the alpha test data used to calibrate our weights. Code and necessary data are accessible under a Creative Commons license at https://github.com/awilson12/risk_scoring
