# Supplementary material for: Quantifying SARS‐CoV‐2 Infection Risk Within the Google/Apple Exposure Notification Framework to Inform Quarantine Recommendations
Source: Risk Anal. 2021 Jun 21;42(1):162–76. doi: 10.1111/risa.13768 (PMC8447042; doi:10.1111/risa.13768)
Supplement: Supplementary file 1 — Supplementary Table I. Parameter values used by app to calculate risk Supplementary Table II. Parameter values used by us to calibrate parameter values in Table 1 Fig. S1. Attenuation data cleaning Fig. S2. Although our dose‐response curve takes the function of an expectation, for low infection probabilities the effect of this is to change the interpretation of the value of λ, which is 3.70 x 10‐6 when using Eq. 5 but 0.3 times this value when using Eq.6 with a distribution of log‐dose with standard deviation corresponding to a 16‐fold difference in dose. [file RISA-42-162-s001.docx]

**Supplemental Materials for**

**Quantifying SARS-CoV-2 infection risk within the Google/Apple exposure notification framework to inform quarantine recommendations**

# Supplementary Table I. Parameter values used by app to calculate risk

| **Parameter** | **Description** | **Distribution or Point Value** | | **References** |
| --- | --- | --- | --- | --- |
| $S$ | Viral shedding rate in arbitrary units that are proportional to viral copies/m^3^ | 10^1^ | 5 days pre- or 8-9 days post-symptom onset | 10-fold range informed by TCID50 measures^1^, timing informed by^1–4^ |
|  |  | 10^1.2^ | 6-7 days post-symptom onset, or asymptomatic within 2-3 days of test |  |
|  |  | 10^1.4^ | 4 days pre- or 5 days post-symptom onset, or asymptomatic within 1 day of test |  |
|  |  | 10^1.6^ | 3 days pre- or 4 days post-symptom onset, or asymptomatic on test day |  |
|  |  | 10^1.8^ | 2 days pre- or 3 days post-symptom onset |  |
|  |  | 10^2^ | 1 day pre- to 2 days post-symptom onset |  |
| $T$_low_, $T$_med_, $T$_high_ | Duration of exposure | Durations for Bluetooth attenuations ≤50dB, 50-60dB, 60-70dB, and >70dB are multiplied by weights 2, 1, 0.5, 0 respectively. | | This study |
| λ | Probability that one viral particle establishes infection × conversion from arbitrary units | 3.70 x 10^-6^ | | Calibrated from secondary attack rate of household contacts = 30%.^5^ |
| Fraction of asymptomatic infections | Higher values lead to longer quarantine | 20% for a population, but depends on age | | ^6^ |
| Incubation period | Days until symptom onset | Probabilities for {0,1,2…} days = {0,4E-05, 0.011842, 0.088541, 0.181965, 0.207344, 0.174797, 0.123761, 0.081488, 0.051057, 0.031469, 0.018734, 0.011235, 0.006786, 0.00422, 0.002518, 0.001626, 0.000978, 0.000592, 0.000364, 0.000231, 0.00014, 0.000093, 0.000062, 0.00004, 0.000025, 0.000017, 0.000011, 0.000008} | | ^7^ |
| Asymptomatic shedding duration | We assume that asymptomatic shedding begins 3 days before what would have been the day of symptom onset if symptomatic, or else immediately upon infection, whichever occurs later, and that shed viral particles are nonviable beyond 12 days | Cumulative probabilities for days {5,6,7…}={0.054054054,0.094594595,0.12162162,0.148648649, 0.189189189,0.21621621,0.256756757,1} | | ^8,9^ |
| Risk threshold | Set by public health agency, cognizant of limitations in current calibration of λ | Benchmark of 0.5% for probability or infection or 0.13% for probability of current or future infectiousness | | This study |

# Supplementary Table II. Parameter values used by us to calibrate parameter values in Table 1

| **Parameter** | **Description** | **Distribution or Point Value** | **References** |
| --- | --- | --- | --- |
| $X$ | Exhalation rate (m^3^/day) | Normal(16.3, 4.15), left-truncated at 9 | ^10^ |
| $A$ | Cross-sectional area of the mouth. Used to calculate the breath velocity, $U$ from $X$ | Uniform(23, 59) (cm^2^) | ^11^ |
| $I_{y}$ | “Lateral intensity” of plume deviation | Uniform(0.08, 0.25) | ^12^ |
| $I_{z}$ | “Vertical intensity” of plume deviation | Uniform(0.03, 0.07) |  |
| $I$ | Inhalation rate (m^3^/day) | Normal(16.3, 4.15), left-truncated at 9 | ^10^ |
| $\rho$ | Distance | Sampled from attenuation-distance dataset to inform weights. | This study |
| φ | Angle between the z-axis and the xy-plane | Used while informing weights. If $\rho$≤ 1m, φ= π/2,If $\rho$ > 1m,φ randomly sampled from Triangular(min= π/4, mode= π/2, max= 3π/4) | 1 m. cutoff for face-to-face interaction informed by ^13^ |
| θ | Angle between x and y axes | Used while informing weights. If $\rho$≤ 1m, θ = 0,If $\rho$ > 1m, θ randomly sampled from Uniform(0, 2π) | 1 m. cutoff for face-to-face interaction informed by ^13^ |


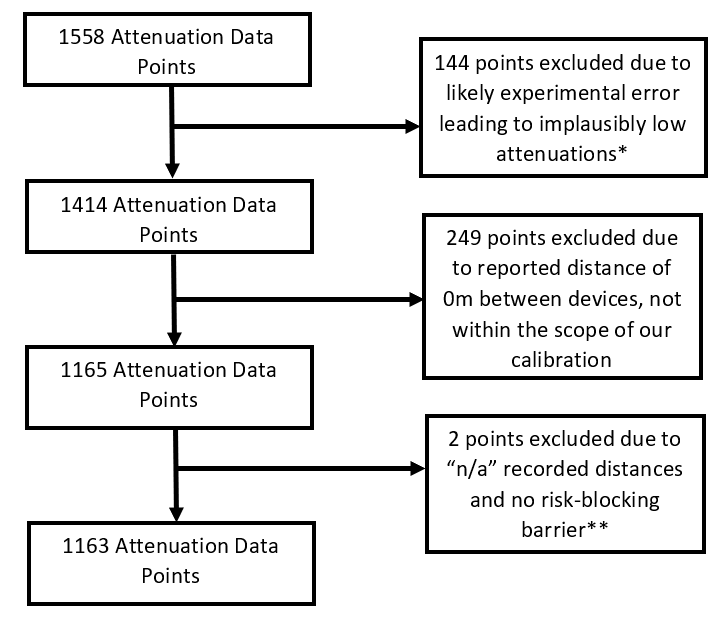


# Figure S1. Attenuation data cleaning

*A few attenuation values were implausibly low, always representing one such increment per device per series of attenuation values corresponding to a single test. We believe this is because these testers used the dropdown menu to turn off Bluetooth rather than going to Settings, and this only disables existing Bluetooth connections, causing an anomalously strong signal to be recorded during the period in which the test was being set up. We manually annotated these, totaling 144 datapoints, and excluded them from further analysis, yielding 1414 datapoints.

**Distance is not needed for the 128 datapoints taken in the presence of a risk-blocking barrier (e.g. closed car doors or walls), so points marking “N/A” for distance were not excluded for any of these.

# 1.0 Variance in Dose

Our dose response curve is actually the probability of infection as a function of the expectation of dose, rather than dose. To consider the effect of variance in dose, we note that while $E\left[ 1-e^{-\lambda D} \right]$ is analytically intractable with respect to a lognormally distributed dose, there is a viable saddle point approximation described by Rojas-Nandayapa (2008).^14^ While Eq. 5.6 in Rojas-Nandayapa (2008) contains a typo, we use the method given to derive

$1-E\left[ e^{-\lambda D} \right]=1-\left[ \frac{1}{\sqrt{1+LW\left( \theta\sigma^{2}e^{\mu} \right)}}exp\left( \frac{LW^{2}\left( \theta\sigma^{2}e^{\mu} \right)+2LW\left( \theta\sigma^{2}e^{\mu} \right)}{2\sigma^{2}} \right) \right],$ (6)

where $LW\left( x \right)$ is the Lambert-W function.

Using this approximation, or the associated importance sampling method^15^ which yields similar results, we can compare the shape for the dose-response curves and consider whether an “effective” $\lambda$ will perform acceptably. Ct counts in Long et al. (2020)^8^ have a standard deviation ~4, representing an upper bound of a 16-fold difference in viral load, because a difference of 1 Ct in PCR measures represents at most a 2-fold difference in underlying viral load, and because individuals are not sampled at exactly comparable times with respect to the timecourse of shedding. However, variance in dose from causes other than infectiousness, e.g. the intimacy of contact, is not included. With a standard deviation representing a 16-fold difference, we find that by using a value 3/10^th^ of $\lambda= 3.70 E-06$, we can super-impose the two curves up to 20% infection probability (Fig. S2).


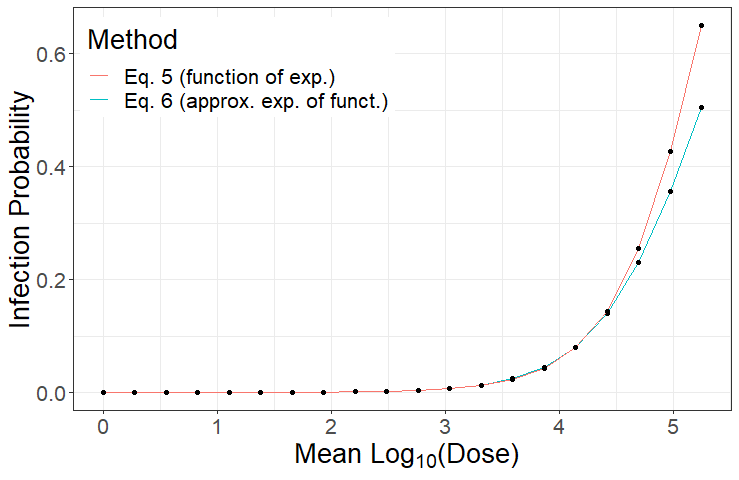


Figure S2. Although our dose-response curve takes the function of an expectation, for low infection probabilities the effect of this is to change the interpretation of the value of $\boldsymbol{\lambda}$, which is 3.70 x 10^-6^ when using Eq. 5 but 0.3 times this value when using Eq.6 with a distribution of log-dose with standard deviation corresponding to a 16-fold difference in dose.

# 2.0 Infectiousness

Infectiousness in the GAEN API is a proxy for the magnitude of viral shedding. In both version 1 and version 2 of the Covid Watch app, it is set on the basis of a simple questionnaire administered to users reporting a positive diagnosis. We use this to inform expected shedding $S$. Infectiousness can be encoded by the 8 “Transmission risk levels” in GAEN version 1, and by the two non-zero “infectiousness” levels in GAEN version 2. By repurposing “report type” metadata associated with Temporary Exposure Keys, 8 levels of infectiousness have also been implemented in Germany’s version 2 Corona-Warn-App,^16,17^ requiring separate calculations for shared key servers that need to be interoperable (Justus Benzler, personal communication).

The first question asked by the Covid Watch app to inform infectiousness is, "What day did your symptoms start"? This question has since been made integral to the function of GAEN version 2. A curve fit to known transmission events suggests peak transmission around the day of symptom onset.^18,19^ Estimates of transmission rates as a function of time relative to symptom onset have been used to estimate infectiousness.^20^ However, infectiousness risk as estimated post-symptom onset from transmission rates might be confounded with behavioral changes with symptom onset, leading to the underestimation of post-symptomatic infectiousness.

A second proxy for infectiousness comes from quantitative polymerase chain reaction (PCR). However, this may reflect non-infectious viral remnants, especially late in the course of disease, where the proportion of culture-positive PCR results tends to decrease.^1,2^ We note that this decline is also expected from a simple dose-response curve, where the probability of culture-positivity decreases as the amount of shedding decreases late in infection, i.e. the decline in infectivity might be quantitative rather than qualitative.

Data in which virus was successfully cultured from patient samples is the clearest metric of post-symptom onset infectivity. Arons et al. ^2^ took prospective samples throughout a nursing home, and were able to culture virus from six days before symptom onset until nine days after symptom onset, with little quantitative trend in shedding rate conditional on a positive test. In hospitalized patients, Wölfel et al. ^3^ were unable to isolate live virus from cultures more than 8 days post symptom onset, despite PCR evidence of high shedding. In one case report, live virus has been isolated 18 days after symptom onset, but this seems to be an outlier.^21^ Bullard et al. ^1^ quantified both TCID50 and PCR for 7 days post symptom onset, and saw an approximately 10-fold decline in infectious dose. We note that culture methods may not be sensitive enough to capture low concentrations.^2^ More studies measuring infectivity in a quantitative manner are needed, particularly in populations that represent a broader base of cases of varying ages and health status. Encouragingly, while our infectiousness settings based on culture data did not agree with initial epidemiological estimates of transmission,^22^ they agreed after corrections to the latter were made.^18^

A final source of information comes from detailed Taiwanese contact tracing^23^, who found a 1.0% symptomatic attack rate (95% CI 0.6-1.6%) for those exposed within five days of symptom onset, and 0% (95% CI 0–0.4%) for those exposed after. Risk from exclusively pre-symptomatic exposure was 0.7% (95% CI 0.2%-2.4%). German contact tracing also points to highest transmission risk around the time of symptom onset.^24^

Here we propose the use of 6 infectiousness levels in the GAEN API, evenly spaced on a log scale between 10 and 100 in arbitrary units, reserving the use of 2 levels for future functionality e.g. regarding superspreaders. Infectiousness levels could also be manipulated for testing purposes, e.g. to help learn, if individuals voluntarily share exposure details centrally, how infectiousness varies in the real world as a function of symptomatic status and timing. We note that most GAEN version apps launched with only one level of infectiousness, and that GAEN version 2 is designed in anticipation of the use of only two the use of only two. While Germany has succeeded in repurposing metadata to provide more, this increases the complexity of interoperability. Given that a systematic 10-fold difference in TCID50 has been observed, supported by epidemiological data, more than 2 levels seem warranted, and making them an intrinsic part of the GAEN system would increase the likelihood of their being used.

Based on a holistic reading of the four sources of evidence described above, we assign the maximum level of 6 from one day pre-symptom onset to two days post-symptom onset. Five days before symptom onset we assign level 1, four days before we assign level 3, three days before level 4, and two days before level 5. Three days after symptom onset we assign level 5, four days after level 4, five days after level 3, 6-7 days after level 2, and 8-9 days after level 1. Our termination at 9 days is based on current CDC guidance.^9^ Pre-symptomatic infectiousness assignments can be further refined in cases when the date of exposure is known, given that the infectious period is longer for longer incubation periods.^19^

For users who report a positive test but no symptoms, there is likely a reason they were tested, and so we ask for the most likely day of exposure, if known. If provided, we assume that shedding did not begin until two days after exposure, at the earliest. We also ask for the date of sampling for the positive test (which can be reported by the healthcare provider rather than the app user) and assume peak shedding at around this time. Subject to the constraint from day of exposure, we assign infectiousness 4 to the day of the test, 3 to the day before and after, and level 2 to dates between 2 and 3 days of the test. There is some evidence that viral shedding is lower in asymptomatic vs. symptomatic cases,^25^ while another study indicates the shedding magnitudes may be similar.^26^ Note that we assume that those with no symptoms at the time they receive a positive test result are asymptomatic rather than pre-symptomatic. We recommend allowing app users to report symptom onset after the fact and trigger a change to previously reported infectiousness. GAEN version 2 TEK revocation functionality makes this possible.

# 3.0 Considerations in recommending and messaging variable quarantine durations

The need for consistent guidance to the public is an important consideration for implementing tailored risk scoring and modified quarantine recommendations. If for the sake of a consistency, a public health authority is not willing to authorize variable quarantine recommendations, as was the case in Arizona when this work was conducted, but only 0 or 14 day quarantines from time of the last individually significant exposure, then the threshold for going into quarantine at all would need to become more strict in order to maintain the same overall risk among the population under quarantine. In other words, retaining the same average probability of current or future infectiousness among the quarantined population would require some exposed individuals to no longer go into quarantine at all, in addition to others lengthening their quarantine out to 14 days. With a binary 0 or 14 day quarantine, the amount by which disease transmission is prevented per day of quarantine will be lower. Considerations are similar for alternative CDC guidelines^27^ for 10 day quarantine or 7 day given a negative test in the last 48 hours.

Alternatively, to avoid mixed messaging regarding quarantine even while the app recommends quarantine of variable duration, one option is to suppress all details about individual exposures from the user’s view, including their date. This has the additional advantage of decreasing the risk that users will be able to guess who exposed them, further preserving privacy.

The app can communicate the risk of infectiousness either as a simple recommendation for which days to quarantine, or also as a quantitative score in order to “game-ify” the process of quarantine and give users positive feedback for each day they succeed in remaining at home until risk falls to a lower level. Further research is needed to assess the most effective messaging strategies. E.g., the app could display both current and projected risk of infectiousness on a simple scale of 1 to 10, so users can see how that risk will fall with each day of quarantine. This visualization might change perceptions. E.g., an individual who wants to comply with a 14-day quarantine, but does not feel able to, might rush out to get groceries before starting their quarantine in earnest, while shedding virus pre-symptomatically. Visualizing projected risk into the future would then give the message that if the exposed individual can only make do for one more day before leaving the home for essentials, that will help, because if they do not develop symptoms, their risk will be lower even after a single day longer. Risk communication in an app could focus on day to day coaxing of this form. Basing quarantine recommendation on a threshold for the expected number of onward transmissions per day is more socially optimal than a threshold on the conditional probability of infectiousness as described here.^28^

Conflicting messages can still arise if manual contact tracers trace an individual who also received an exposure notification. In this case, it is likely that the two recommend different end dates for quarantine. While this is to be expected from our procedure for recommending variable quarantine durations, we note that even if the app were to issue 14 day quarantine recommendations only, it could still arise because the individual has been exposed more than once, on different days, and the manual contact tracer is following up an infected individual who may not have used the app. Until there is reliable data on app performance, we recommend that the manual contact tracer’s protocol should override whatever the app says. Should the app turn out to perform well, an alternative procedure might eventually be to go with whichever protocol recommends the longer quarantine. An intermediate possibility is for the manual contact tracer to ask for exposure notification details, to determine whether it may be a different exposure to the one being manually traced. There may also be conflicts in protocols for the timing of testing.

Note that with symptom onset sometimes as early as two days after exposure, and given the possibility of pre-symptomatic shedding, and the possibility of confusion regarding who infected whom, we currently ignore the possibility that shedding might not yet have begun. Current testing turnaround times are mostly long, making this reasonable. However, if same-day tests become more widely available, our approach could be extended to directly communicate the risk of current infectiousness, whose calculation is described in Petrie & Masel (manuscript in prep.)^29^, rather than as is currently the case, the risk of current or future infectiousness. A significantly lower risk of infectiousness will be present on the day of exposure and perhaps also the day after. Delays in going into public to prepare for a long quarantine could inadvertently lead to pushing individuals past the latent period before they go into public; displaying a full projected timeline of the projected risk of infectiousness could avert this, at the risk of significantly more complex messaging than “stay home until Friday”. The use of current infectiousness would prevent the app from occasionally recommending quarantines of less than 5 days when initial risk is already very near the threshold.

# References

1. Bullard J, Dust K, Funk D, et al. Predicting infectious SARS-CoV-2 from diagnostic samples. *Clin Infect Dis*. May 2020. doi:10.1093/cid/ciaa638

2. Arons MM, Hatfield KM, Reddy SC, et al. Presymptomatic SARS-CoV-2 infections and transmission in a skilled nursing facility. *N Engl J Med*. 2020;382(22):2081-2090. doi:10.1056/NEJMoa2008457

3. Wölfel R, Corman VM, Guggemos W, et al. Virological assessment of hospitalized patients with COVID-2019. *Nature*. 2020;581(7809):465-469. doi:10.1038/s41586-020-2196-x

4. Ashcroft P, Huisman JS, Lehtinen S, et al. COVID-19 infectivity profile correction. *Swiss Med Wkly*. 2020;(150):w20336. doi:10.4414/smw.2020.20336

5. Curmei M, Ilyas A, Evans O, Steinhardt J. Estimating household transmission of SARS-CoV-2. *medRxiv*. 2020. doi:10.1101/2020.05.23.20111559

6. Buitrago-Garcia DC, Egli-Gany D, Counotte MJ, et al. Asymptomatic SARS-CoV-2 infections: a living systematic review and meta-analysis, version 3. *medRxiv*. 2020. doi:10.1101/2020.04.25.20079103

7. Lauer SA, Grantz KH, Bi Q, et al. The incubation period of coronavirus disease 2019 (CoVID-19) from publicly reported confirmed cases: Estimation and application. *Ann Intern Med*. 2020;172(9):577-582. doi:10.7326/M20-0504

8. Long Q-X, Tang X-J, Shi Q-L, et al. Clinical and immunological assessment of asymptomatic SARS-CoV-2 infections. *Nat Med*. 2020;26(8):1200-1204. doi:10.1038/s41591-020-0965-6

9. Centers for Disease Control and Prevention. Duration of Isolation & Precautions for Adults. https://www.cdc.gov/coronavirus/2019-ncov/hcp/duration-isolation.html#cecommendations. Published 2020. Accessed July 27, 2020.

10. U.S. Environmental Protection Agency. *Exposure Factors Handbook 2011 Edition (EPA/600/R-09/052F)*. Washington, DC; 2011. https://cfpub.epa.gov/ncea/risk/recordisplay.cfm?deid=236252.

11. Leckie JO, Naylor KA, Canales RA, et al. *Quantifying Children’s Microlevel Activity Data from Existing Videotapes” by Exposure Research Group at Stanford University for the U.S. Environmental Protection Agency*.; 2000. https://cfpub.epa.gov/ols/catalog/advanced_full_record.cfm?&FIELD1=AUTHOR&INPUT1=FERGUSON AND C. AND R.&TYPE1=ALL&LOGIC1=AND&COLL=&SORT_TYPE=MTIC&item_count=5.

12. Western Engineering. Self-study notes - GAUSSIAN PLUMES. https://www.eng.uwo.ca/people/esavory/Gaussian plumes.pdf. Accessed June 7, 2020.

13. Zhang N, Su B, Chan PT, Miao T, Wang P, Li Y. Infection spread and high-resolution detection of close contact behaviors. *Int J Environ Res Public Health*. 2020;17(4):1445. doi:10.3390/ijerph17041445

14. Rojas-Nandayapa L. Risk Probabilities: Asymptotics and Simulation. 2008. PhD Dissertation. Department of Mathematical Sciences, University of Aarhus.

15. Asmussen S, Jensen JL, Rojas-Nandayapa L. On the Laplace Transform of the Lognormal Distribution. *Methodol Comput Appl Probab*. 2016;18:441-458. doi:10.1007/s11009-014-9430-7

16. Klingbeil T. corona-warn-app / cwa-documentation / images / risk_calculation / server_encoding.pdf. GitHub. https://github.com/corona-warn-app/cwa-documentation/blob/master/images/risk_calculation/server_encoding.pdf. Published 2020. Accessed January 6, 2021.

17. Klingbeil T. corona-warn-app / cwa-documentation / images / risk_calculation / client_interpretation.pdf. GitHub. https://github.com/corona-warn-app/cwa-documentation/blob/master/images/risk_calculation/client_interpretation.pdf. Published 2020. Accessed January 6, 2021.

18. He X, Lau EHY, Wu P, et al. Author Correction: Temporal dynamics in viral shedding and transmissibility of COVID-19. *Nat Med*. 2020;26:1491-1493. doi:10.1038/s41591-020-1016-z

19. Ferretti L, Ledda A, Wymant C, et al. The timing of COVID-19 transmission. *medRxiv*. 2020. doi:10.1101/2020.09.04.20188516

20. CWA Team. corona-warn-app / cwa-documentation / transmission_risk.pdf. GitHub. https://github.com/corona-warn-app/cwa-documentation/blob/master/transmission_risk.pdf. Published 2020. Accessed January 6, 2021.

21. Liu W-D, Chang S-Y, Wang J-T, et al. Prolonged virus shedding even after seroconversion in a patient with COVID-19. *J Infect*. 2020;81(2):318-356. doi:10.1016/j.jinf.2020.03.063

22. He X, Lau EHY, Wu P, et al. Temporal dynamics in viral shedding and transmissibility of COVID-19. *Nat Med*. 2020;26(5):672-675. doi:10.1038/s41591-020-0869-5

23. Cheng HY, Jian SW, Liu DP, Ng TC, Huang WT, Lin HH. Contact tracing assessment of COVID-19 transmission dynamics in Taiwan and risk at different exposure periods before and after symptom onset. *JAMA Intern Med*. 2020;180(9):1156-1163. doi:10.1001/jamainternmed.2020.2020

24. Böhmer MM, Buchholz U, Corman VM, et al. Investigation of a COVID-19 outbreak in Germany resulting from a single travel-associated primary case: a case series. *Lancet Infect Dis*. 2020;20(8):920-928. doi:10.1016/S1473-3099(20)30314-5

25. McDonald J. Unpacking WHO’s Asymptomatic COVID-19 Transmission Comments. FactCheck.org. https://www.factcheck.org/2020/06/unpacking-whos-asymptomatic-covid-19-transmission-comments/. Published 2020. Accessed June 16, 2020.

26. Lavezzo E, Franchin E, Ciavarella C, et al. Suppression of a SARS-CoV-2 outbreak in the Italian municipality of Vo’. *Nature*. 2020;584(7821):425-429. doi:10.1038/s41586-020-2488-1

27. Centers for Disease Control and Prevention. Options to Reduce Quarantine for Contacts of Persons with SARS-CoV-2 Infection Using Symptom Monitoring and Diagnostic Testing. https://www.cdc.gov/coronavirus/2019-ncov/more/scientific-brief-options-to-reduce-quarantine.html. Published 2020. Accessed January 10, 2020.

28. Petrie J, Masel J. The economic value of quarantine is higher at lower case prevalence, with quarantine justified at lower risk of infection. *medRxiv*. 2020. doi:10.1101/2020.11.24.20238204

29. Petrie J, Masel J. Quarantine Optimization. 2021:In preparation.
